# Determinants of Social Distancing Among South Africans From 12 Days Into the COVID-19 Lockdown: A Cross Sectional Study

**DOI:** 10.3389/fpubh.2021.632619

**Published:** 2021-05-24

**Authors:** Ronel Sewpaul, Musawenkosi Mabaso, Natisha Dukhi, Inbarani Naidoo, Noloyiso Vondo, Adlai Steven Davids, Tholang Mokhele, Sasiragha Priscilla Reddy

**Affiliations:** ^1^Human and Social Capabilities Division, Human Sciences Research Council, Pretoria, South Africa; ^2^Faculty of Health Sciences, Nelson Mandela University, Port Elizabeth, South Africa; ^3^eResearch Knowledge Centre, Human Sciences Research Council, Pretoria, South Africa

**Keywords:** COVID-19, South Africa, lockdown, social determinants of health (MeSH), stay at home directive, physical distancing and research, social distancing behaviour

## Abstract

**Introduction:** Social or physical distancing has been an effective measure for reducing the spread of COVID-19 infections. Investigating the determinants of adherence to social distancing can inform public health strategies to improve the behaviour. However, there is a lack of data in various populations. This study investigates the degree to which South Africans complied with social distancing during the country's COVID-19 lockdown and identifies the determinants associated with being in close contact with large numbers of people.

**Materials and Methods:** Data was collected from a South African national online survey on a data free platform, supplemented with telephone interviews. The survey was conducted from 8 to 29 April 2020. The primary outcome was the number of people that participants came into close contact with (within a 2-metre distance) the last time they were outside their home during the COVID-19 lockdown. Multivariate multinomial regression investigated the socio-demographic, psychosocial and household environmental determinants associated with being in contact with 1–10, 11–50 and more than 50 people.

**Results:** Of the 17,563 adult participants, 20.3% reported having not left home, 50.6% were in close physical distance with 1–10 people, 21.1% with 11–50 people, and 8.0% with >50 people. Larger household size and incorrect knowledge about the importance of social distancing were associated with being in contact with >50 people. Male gender, younger age and being in the White and Coloured population groups were significantly associated with being in contact with 1–10 people but not with larger numbers of people. Employment, at least secondary school education, lack of self-efficacy in being able to protect oneself from infection, and moderate or high risk perception of becoming infected, were all associated with increased odds of close contact with 1–10, 11–50, and >50 people relative to remaining at home.

**Conclusion:** The findings identify subgroups of individuals that are less likely to comply with social distancing regulations. Public health communication, interventions and policy can be tailored to address these determinants of social distancing.

## Introduction

The coronavirus, COVID-19, that was first discovered in China in December 2019, continues to pose a significant global public health threat. At the end of October 2020, there were over 43 million confirmed cases and 1.1 million COVID-19 related deaths globally (1). SARS-CoV-2, the strain of coronavirus that causes COVID-19, can be spread by respiratory droplets from person to person when in close contact (2) while airborne transmission is also plausible (3). In the absence of pharmaceutical interventions, governments have promoted behavioural change measures such as social or physical distancing, wearing of face masks, and frequent hand washing or sanitising, to reduce viral transmission (4–6).

Social distancing refers to maintaining at least a metre distance between individuals and the avoidance of crowded gatherings with the potential for close contact (4). It has been demonstrated that social distancing resulted in reduced COVID-19 infections and transmissions (5, 7–9). In the United States, a 50% decrease in non-essential business visits was associated with a 45% decrease in transmissibility (9). In order to reduce the number of social contacts and thereby slow the viral spread, countries have introduced regulations such as closing of shops, educational institutions, and restaurants, prohibition of mass gatherings and public events, and work from home directives (10). Data from modelling and observational studies have shown that social distancing interventions, such as bans on mass gatherings, school and workplace closures and movement restrictions, are associated with lower incidence of COVID-19 infections and reduced mortality (11–15). Longitudinal analysis of outbreak epicentres in 37 OECD countries during the first pandemic wave found that a 1-day delay in the mass gatherings ban and a 1-day delay in school closures were associated with increases in COVID-19 cumulative mortality of 6.9% and 4.4%, respectively (15).

In South Africa, the first COVID-19 cases were discovered in early March 2020. To promote social distancing and minimise COVID-19 spread, measures to reduce interpersonal interactions were introduced in mid-March and a national 21-day lockdown was imposed to begin on 27 March 2020 (16). South Africans were required to remain at home and were only allowed out during strictly controlled conditions such as purchasing of food, medicines and other essentials; to seek medical care or to collect a social grant. The lockdown created an opportunity to break COVID-19 transmission, as a 14-day incubation period exists during which the infection symptoms can become distinct. The regulations were accompanied by public health advice on hygiene and keeping a 1–2 metre distance from others when outside of the home. Under a risk adjusted strategy, the lockdown was extended by 2 weeks to 30th April 2020, allowing for the economy to reopen partially. The behavioural practises of wearing a face mask and disinfecting surfaces were made mandatory. From 1 to 31 May the country transitioned to an “Alert level 4” lockdown, and then from 1 June to 17 August to “Alert level 3,” with more economic sectors reopening at each stage. Following a second wave of infections during November 2020 to January 2021, the country is at level 1 lockdown with economic sectors having reopened, and therefore with the need for strict social distancing measures to be heeded (17).

Despite governmental regulations to combat the outbreak, the degree of success in mitigating the spread of COVID-19 is largely dependent on public adherence to social distancing and other behavioural interventions that were regulated. It was estimated that if social distancing among South Africans declined by 2% there could be a 23% increase in cases (18). Further, the South African COVID-19 Modelling Consortium (19) indicated that adherence to social distancing and other public health regulations during the lockdown in the first wave could result in delaying the epidemic peak by 2–3 months, allowing time for the health system to adequately prepare. It is unclear for how long social distancing measures will need to be practised as subsequent waves of COVID-19 may emerge.

Understanding the determinants of adherence to social distancing will inform targeted and tailored public health interventions to address the behaviour. Studies have shown that transmission reducing behaviours like social distancing during infectious disease outbreaks were influenced by increased risk perceptions (6, 20, 21), self-efficacy to implement these behaviours (21, 22) and high knowledge about prevention and transmission (23–26). In addition, gender, older age, income and education were also associated with adherence to social distancing (22, 25, 27–29). However, evidence from low- and middle-income countries (LMICs) is scarce. COVID-19 research priorities need to factor in social determinants of health, as compliance with distancing behaviours are challenging for individuals with adverse social determinants such as crowded living conditions (30). Notably, a large proportion of South Africans live in crowded settlements where large numbers of people live in small homes and families share water and sanitation services.

Using data from a nationwide population-based survey, this study investigates the degree to which South Africans complied with social distancing during the country's COVID-19 lockdown, as measured by the number of people that they came into close contact with the last time they were outside their homes. It further identifies the socio-demographic, psychosocial and household environmental determinants associated with being in contact with 1–10, 11–50 and more than 50 people, where contact with more than 50 people is indicative of crowding. We hypothesised that participants from lower socioeconomic environments, from households with large numbers of people and who use public transport would report being in contact with larger numbers of people. At the time of writing this paper, the role of psychosocial and environmental factors on the number of close contacts had not been previously assessed in South Africa. The findings will allow us to confirm if the patterns of association with knowledge, self-efficacy, risk perception and demographic factors found in studies from high income countries persist in South Africa. The findings can inform public health and policy directives to improve adherence to social distancing.

## Materials and Methods

A rapid online survey, supplemented with telephone facilitated interviews, was conducted during the COVID-19 lockdown in South Africa. South African adults in all nine provinces were eligible to participate. The invitation links to participate in the survey were widely distributed on a data-free mobile messaging platform and via numerous communication and media channels, including social media, national and local radio, national television news, email, local websites and a wide network of strategic partners in government, education, faith-based and community organisations, non-profit organisations and the private sector. All participants were encouraged to share the survey link. The data-free mobile messaging platform allowed participants to complete the survey without incurring data costs. The platform was chosen because it has a large user-base of more than four million South Africans and can be downloaded from all application stores. The online questionnaire and telephonic interviews were available in English, Afrikaans, Sepedi, isiZulu, and isiXhosa.

Telephone interviews supplemented the online survey approach, to include participants that may not have responded to an online survey. Interviewers were trained by the research team in obtaining informed consent and telephone interview procedures. The team of interviewers were collectively fluent in the five languages in which the survey could be completed. A list of telephone numbers of over one million people in predominantly densely populated areas such as informal settlements and townships (urban residential settlements) was used to recruit participants in the telephone survey, where 3,602 people from the list were telephonically contacted and 2,682 participated.

The questionnaire was developed in consultation with epidemiologists, behavioural and public health scientists. Discussions were held to identify key thematic areas that would provide insight into the attitudes and behaviours among the general South African population. Questionnaire development occurred as South Africa begun its lockdown and when very little was known about COVID-19 local transmission in the country. Questionnaire development was informed by previous work on public reactions to the pandemic (31, 32) and from a South African survey conducted a few weeks prior (33). Consultations with stakeholders in scientific and civil society networks were used to further refine the questionnaire. The thematic areas identified and included in the questionnaire were demographic and household characteristics, knowledge about COVID-19 and preventative measures, public concerns about the pandemic, self-efficacy about the ability to protect oneself from infection, risk perception, personal experience with testing and screening, attitudes toward lockdown measures, travel behaviour, physical distancing, access to essentials like food, water, sanitation, healthcare and chronic medicines, and the socio-economic impact of the lockdown measures. Five-point Likert scales were used for the questions on self-efficacy, risk perception and socio-economic impact. The questionnaire comprised 55 items of which 54 were close ended. The survey was conducted during 8–29 April 2020. The online survey was available to complete during 8–24 April and the telephone interviews were conducted during 8–29 April, as telephone data collections required a longer time to complete. The survey period corresponded to the 2nd−4th week (12th−33rd day) of the state-implemented “Alert level 5” lockdown period.

### Ethical Procedures

Ethical approval to conduct the study was received from the Human Sciences Research Council Research Ethics Committee (HSRC REC) (Protocol number: REC 5/03/20), which is aligned with the principles expressed in the Declaration of Helsinki. Informed consent was obtained from participants before they were directed to the survey questions. Participants were informed of voluntary participation, the anonymity of their responses, and the option to withdraw from the survey at any time.

### Measures

The dependent variable was derived from the question “The last time you were away from home, how many people did you come into close contact with? (within 2 metres),” and the response had 6 options (have not left home, 1–3, 4–10, 11–20, 21–50, and more than 50 people).

The independent variables were classified into socio-demographic variables, psychosocial determinants of behaviour, household environmental and living conditions and economic capability. The questions analysed in the current paper are presented in Supplementary Table 1. The socio-demographic variables considered were gender, age, population group, residential community type, education and employment, where population group was reported in consistency with Statistics South Africa's standard classification categories (34). Variables measuring psychosocial determinants of behaviour were knowledge about behaviours to prevent COVID-19 transmission, feeling that the lockdown was unnecessary and being angered by it, self-efficacy in protecting oneself from infection, and risk perception of becoming infected. Self-efficacy in protecting oneself from infection was evaluated by participants' agreement or disagreement with the statement “I am confident that I can prevent myself from getting COVID-19.” Risk perception was assessed by a single item asking participants to rate their level of personal risk of becoming infected with the virus, with five response options ranging from very high risk to very low risk. The two knowledge items were assessed by participants affirmative responses to the statements “I can prevent myself from becoming infected with the Coronavirus (COVID-19) by staying away from people who are infected” and “I can prevent myself from becoming infected with the Coronavirus (COVID-19) by staying 2 metres away from another person,” with “Yes” or “No” response options. Agreement with the statement “The lockdown was unnecessary and has made me angry” assessed feelings about the lockdown. The variables related to household environmental conditions were whether participants lived in a household that shared water facilities with other households, the number of household members, and access to food during the lockdown. Economic capability referred to the perceived financial difficulty as a result of the COVID-19 lockdown. It was calculated as a sum score of four items, related to feeling that the lockdown was making it difficult to earn their income; to keep their job; would make it difficult to feed their family; and to pay their bills or debts. Each item was measured on a 5-point Likert scale, with options ranging from 1 (strongly disagree) to 5 (strongly agree). The Cronbach's alpha for the four items was 0.91, demonstrating high inter-item reliability. Lower values of the composite score indicated higher perceived financial difficulty. The sum score was grouped into high, moderate and low using the 25th and 75th percentiles. The selection of independent variables was informed by the literature, the Health Belief Model (35) and the Social Determinants of Health (36).

### Statistical Analysis

The data were benchmarked using the South African adult mid-year population estimates by age, race, sex, and province (34) to increase generalizability of the estimates to a national level. All analyses were conducted in Stata 15.0 (Stata Corporation, College Station, Texas, USA). The “svy” command was used to incorporate benchmarking weights into the analysis. Summary statistics were used to describe the characteristics of the sample. Pearson Chi-square tests were used to detect significant differences in estimates of categorical variables.

Preliminary cross-tabulations and multinomial regressions showed that for the majority of independent variables, the patterns of association (the relative risk ratios) for coming into contact with 1–3 people were similar to those of coming into contact with 4–10 people. A similar finding was observed between the categories of 11–20 and 21–50 people. The response options for close contacts were therefore recoded into four categories; 1–10 people, 11–50 and >50 and did not leave home, in order to facilitate meaningful comparisons across a smaller number of categories.

A multivariate multinomial logistic regression analysis was used to determine factors associated with the numbers of people that participants came into close contact with, where “None/did not leave home” was used as the reference category. All independent variables that had a significant univariate association with the outcome variable, as measured by the Chi-square tests, were used in the multivariate multinomial model. Odds ratios with 95% confidence intervals (CIs) were used to assess the strength and direction of the associations. All statistical tests were considered significant at a *p*-value < 0.05.

## Results

### Characteristics of the Weighted Sample

Table 1 shows that of the sample of 17 563 individuals, more than half were female (53%); 70.1% were 25–59 years old; the majority were African (77.9%) and had matric (grade 12) or higher-level education (79.6%); 36.6% had full time employment, 37.2% were unemployed, 33.9% resided in townships and 21% lived in rural traditional tribal areas.

**Table 1:** Characteristics of the study sample.

**Variables**	**Total**	**%**	**95% CI**
**Socio-demographic**			
Gender			
Female	10,769	53.0	51.9–54.1
Male	6,614	47.0	45.9–48.1
Other	180	<0.1	0.0–0.0
Age (years) (Mean, S.E.)		40.3	0.21
18–24	2,850	15.2	14.5–15.8
25–59	13,160	70.1	69.0–71.2
60–69	1,107	8.7	8.0–9.5
≥70	341	6.0	5.2–7.0
Population group			
African	8,689	77.9	77.2–78.5
White	6,018	10.4	10.1–10.8
Coloured	1,877	8.6	8.2–9.1
Indian/Asian	979	3.1	2.8–3.3
Residential community			
City	2,433	10.2	9.6–10.8
Suburb	7,263	28.5	27.5–29.4
Township	4,072	33.9	32.9–34.9
Informal settlement	622	4.3	4.0–4.8
Rural (Traditional tribal area)	2,548	21.0	20.2–22.0
Farm	625	2.1	1.8–2.3
Highest education level			
Less than secondary	557	6.0	5.4–6.6
Secondary	2,301	14.4	13.6–15.1
Matric or higher	14,705	79.6	78.7–80.5
Employment			
Employed full time	7,152	36.6	35.6–37.6
Employed informally/part time	1,674	9.8	9.2–10.4
Student	1,418	7.9	7.4–8.4
Unemployed	5,387	37.2	36.1–38.3
Self employed	1,932	8.6	8.0–9.2
**Psychosocial determinants of behaviour**			
Correct knowledge that staying away from people who are infected can prevent COVID-19 infection	16,752	96.8	96.4–97.2
Correct knowledge that staying 2 metres away from another person can prevent COVID-19 infection	16,521	95.6	95.1–96.0
The lockdown was unnecessary and has made me angry	1,619	11.7	11.0–12.5
**Self-efficacy - I am confident in preventing myself from getting COVID-19**			
Agree	13,626	82.6	81.8–83.4
Neutral	2,901	13.7	13.0–14.5
Disagree	897	3.6	3.3–4.0
**Risk perception of becoming infected**			
Low	8,127	45.0	44.0–46.1
Moderate	5,570	29.3	28.4–30.3
High	3,866	25.6	24.7–26.6
**Household environment and living conditions**			
Ability to get food to your household during the lockdown			
We can buy from a shop within walking distance from my house	4,148	25.7	24.8–26.6
We can buy from a shop, which I reach using a taxi/bus	1,949	15.3	14.5–16.1
We can buy from a shop, which I reach using my car	7,922	35.8	34.8–36.9
Do not have enough money to buy food during the lockdown	3,508	23.2	22.4–24.1
Share water facilities with other households	4,074	26.6	25.6–27.5
Household size (Mean, S.E., range)	4.8	0.03	1–20
**Economic capability**			
Perceived COVID-19 related financial difficulty			
High	4,006	26.8	25.8–27.8
Moderate	7,414	49.0	47.8–50.2
Low	3,382	24.2	23.1–25.3

More than 95% reported correct knowledge that staying away from infected people as well as maintaining a 2-metre distance between other people can prevent COVID-19 (Table 1). A quarter (25.6%) perceived themselves at high risk of infection, 82.6% reported self-efficacy in protecting themselves from COVID-19 infection, and 11.7% felt that the lockdown was unnecessary and had made them angry. Just under one quarter (23.2%) did not have enough money to buy food during lockdown and over one quarter (26.8%) had perceived financial difficulty during lockdown. The average household size was 4.8 people and 26.6% lived in households that shared their water sources with other households.

### Numbers of Close Contacts

Table 2 shows that a fifth of participants (20.3%, 95% CI: 19.4–21.2) reported not being within a 2-metre distance from anyone because they had not left home, 50.6% (49.5–51.7) had come into close physical distance with 1–10 people on the last occasion that they were away from home, 21.1% (20.2–22.0) with 11–50 people, and 8.0% (7.4–8.6) with more than 50 people.

**Table 2:** The number of close contacts when outside the home by socio-demographic characteristics and behavioural determinants.

	**Number of people in close contact with (within a 2-metre distance)**								
	**the last time the participant was away from home**								
	**Did not leave home**		**1–10 people**		**11–50 people**		**>50 people**		
	**%**	**95% CI**	**%**	**95% CI**	**%**	**95% CI**	**%**	**95% CI**	***p*-value**
Total	20.3	(19.4–21.2)	50.6	(49.5–51.7)	21.1	(20.2–22.0)	8.0	(7.4–8.6)	
**Socio-demographic**									
Gender									
Female	21.5	(20.3–22.7)	48.8	(47.4–50.1)	21.3	(20.2–22.5)	8.4	(7.6–9.3)	0.004
Male	19.0	(17.6–20.4)	52.7	(51.0–54.4)	20.7	(19.3–22.2)	7.6	(6.8–8.5)	
Other	17.8	(12.9–24.1)	55.6	(48.2–62.6)	21.1	(15.8–27.7)	5.6	(3.0–10.0)	
Age group (years)									
18–24	20.6	(18.9–22.5)	53.7	(51.5–56.0)	18.3	(16.6–20.1)	7.3	(6.3–8.6)	0.021
25–59	19.2	(18.3–20.2)	50.5	(49.4–51.7)	21.7	(20.8–22.7)	8.5	(7.9–9.2)	
60–69	22.6	(18.9–26.9)	48.7	(44.0–53.4)	22.3	(18.4–26.7)	6.4	(4.5–9.1)	
≥70	28.7	(22.2–36.3)	46.2	(38.6–54.1)	18.6	(12.5–26.7)	6.5	(3.4–11.9)	
Population group									
African	22.5	(21.4–23.6)	46.8	(45.5–48.1)	21.6	(20.5–22.8)	9.1	(8.3–9.8)	<0.001
White	10.8	(9.7–12.0)	68.1	(66.4–69.7)	18.2	(16.9–19.5)	3.0	(2.5–3.6)	
Coloured	14.2	(12.6–16.1)	62.3	(59.7–64.9)	17.9	(15.9–20.0)	5.6	(4.5–6.8)	
Indian/Asian	13.8	(11.4–16.7)	55.1	(51.3–58.9)	25.3	(22.1–28.8)	5.7	(4.4–7.4)	
Community of residence									
City	16.1	(13.8–18.8)	60.4	(57.3–63.4)	17.7	(15.5–20.2)	5.7	(4.4–7.4)	<0.001
Suburb	13.0	(11.6–14.5)	60.4	(58.4–62.3)	21.5	(19.9–23.1)	5.2	(4.4–6.1)	
Township	21.1	(19.6–22.6)	47.2	(45.4–49.0)	22.1	(20.6–23.8)	9.6	(8.5–10.8)	
Informal settlement	19.8	(16.4–23.8)	49.0	(44.4–53.6)	20.1	(16.8–23.8)	11.1	(7.9–15.3)	
Rural (Traditional tribal area)	31.3	(28.9–33.8)	37.7	(35.1–40.3)	20.8	(18.6–23.2)	10.2	(8.9–11.7)	
Farm	17.6	(13.4–22.7)	59.7	(53.4–65.7)	18.6	(14.5–23.6)	4.1	(2.0–8.1)	
Highest educational level									
Less than secondary	45.4	(40.0–50.9)	31.1	(26.0–36.8)	15.2	(11.9–19.2)	8.3	(5.9–11.6)	<0.001
Secondary	27.8	(25.3–30.5)	43.8	(41.1–46.6)	18.3	(16.3–20.6)	10.0	(8.2–12.2)	
Matric or higher	17.1	(16.2–18.0)	53.3	(52.1–54.5)	22.0	(21.0–23.0)	7.6	(7.0–8.3)	
Employment									
Employed full time	13.0	(11.9–14.2)	52.7	(51.1–54.3)	24.6	(23.2–26.1)	9.7	(8.7–10.8)	<0.001
Employed informal/part time	19.1	(16.7–21.7)	50.1	(47.0–53.3)	22.4	(19.9–25.1)	8.4	(6.8–10.2)	
Student	21.6	(19.1–24.4)	49.3	(46.1–52.6)	19.3	(16.9–22.0)	9.7	(7.7–12.2)	
Unemployed	28.1	(26.3–29.9)	47.5	(45.6–49.5)	18.1	(16.5–19.9)	6.3	(5.4–7.3)	
Self employed	18.0	(15.4–20.9)	56.8	(53.2–60.3)	18.5	(15.8–21.6)	6.7	(5.0–8.8)	
**Psychosocial factors**									
Correct knowledge that staying away from people who are infected can prevent COVID-19 infection									
No	18.1	(13.4–24.1)	40.1	(34.2–46.4)	25.4	(20.4–31.2)	16.3	(11.8–22.2)	<0.001
Yes	20.2	(19.3–21.1)	51.0	(49.9–52.1)	21.1	(20.2–22.0)	7.8	(7.2–8.4)	
Correct knowledge that staying 2 metres away from another person can prevent COVID-19 infection									
No	16.4	(12.9–20.7)	47.7	(42.5–52.9)	22.7	(18.9–26.9)	13.2	(10.2–17.0)	0.001
Yes	20.1	(19.2–21.1)	50.9	(49.8–52.0)	21.2	(20.2–22.1)	7.8	(7.2–8.4)	
Thought that the lockdown was unnecessary and were angered by it									
No	18.9	(17.9–19.9)	51.5	(50.2–52.7)	22.1	(21.0–23.2)	7.6	(7.0–8.3)	<0.001
Yes	28.8	(25.7–32.1)	42.6	(39.4–46.0)	17.2	(15.0–19.7)	11.4	(9.5–13.6)	
Self-efficacy in preventing one's self from COVID-19 infection									
Agree	22.4	(21.4–23.5)	50.9	(49.7–52.1)	19.4	(18.4–20.4)	7.3	(6.7–8.0)	<0.001
Unsure	9.8	(8.3–11.5)	50.2	(47.2–53.1)	29.6	(26.8–32.5)	10.4	(8.8–12.3)	
Disagree	11.2	(8.3–14.8)	46.9	(42.0–51.9)	27.2	(23.0–32.0)	14.7	(11.4–18.7)	
Risk perception									
Low	25.9	(24.5–27.3)	50.8	(49.2–52.4)	17.9	(16.6–19.3)	5.4	(4.8–6.1)	<0.001
Moderate	13.6	(12.1–15.2)	54.1	(52.2–56.1)	25.2	(23.5–27.0)	7.1	(6.1–8.2)	
High	18.2	(16.6–20.0)	46.3	(44.1–48.5)	21.8	(20.1–23.5)	13.8	(12.2–15.4)	
**Environmental /household living conditions**									
Ability to get food to your household during the lockdown									
Can buy from a shop, which I reach using my car	13.6	(12.2–15.0)	55.5	(53.6–57.4)	24.1	(22.4–25.9)	6.8	(5.9–7.8)	
Can buy from a shop within walking distance from my house	21.1	(19.3–22.9)	52.5	(50.4–54.5)	19.3	(17.8–21.0)	7.2	(6.1–8.4)	<0.001
Can buy from a shop, which I reach using a taxi/bus (public transport)	22.1	(19.8–24.5)	41.9	(39.2–44.7)	22.9	(20.6–25.3)	13.1	(11.3–15.2)	
Do not have enough money to buy food during the lockdown	28.4	(26.6–30.3)	47.1	(45.0–49.1)	16.9	(15.5–18.5)	7.6	(6.6–8.7)	
Do you share water facilities with other households									
Yes	22.2	(20.5–23.9)	47.6	(45.6–49.7)	21.0	(19.4–22.7)	9.2	(8.1–10.5)	0.001
No	19.6	(18.6–20.7)	51.7	(50.5–53.0)	21.1	(20.0–22.2)	7.6	(6.9–8.3)	
Household size									
1–5 people	19.5	(18.5–20.5)	52.3	(51.0–53.6)	20.9	(19.8–22.0)	7.4	(6.7–8.1)	<0.001
6–10 people	21.9	(20.0–24.0)	47.6	(45.5–49.8)	21.7	(19.9–23.6)	8.8	(7.6–10.1)	
≥10 people	22.3	(18.6–26.5)	42.7	(38.2–47.3)	21.8	(18.3–25.8)	13.2	(9.9–17.3)	
**Economic capability**									
Perceived COVID-19 related financial difficulty									
High	22.2	(20.5–24.1)	51.2	(49.1–53.3)	18.9	(17.4–20.5)	7.7	(6.7–8.9)	<0.001
Moderate	20.0	(18.6–21.5)	51.2	(49.5–52.9)	21.1	(19.6–22.6)	7.7	(6.9–8.7)	
Low	16.6	(14.6–18.8)	47.8	(45.1–50.4)	26.2	(23.9–28.7)	9.4	(8.0–11.1)	

The number of people that participants were in close distance with varied significantly with all the independent variables. The percentage who stayed home and did not come into close distance with others was higher among residents of rural traditional communities (31.3%), Africans (22.5%), participants who did not complete secondary school (45.4%), unemployed participants (28.1%), those with low risk perceptions (25.9%) and those who reported being unable to afford food during the lockdown (28.4%). Having been in close proximity with >50 people was highest among residents of informal settlements, rural traditional areas, and townships; among those with incorrect knowledge about social distancing as a preventive measure, and among those with low self-efficacy to protect themselves from infection.

### Factors Associated With Close Contacts

**Figure 1:**
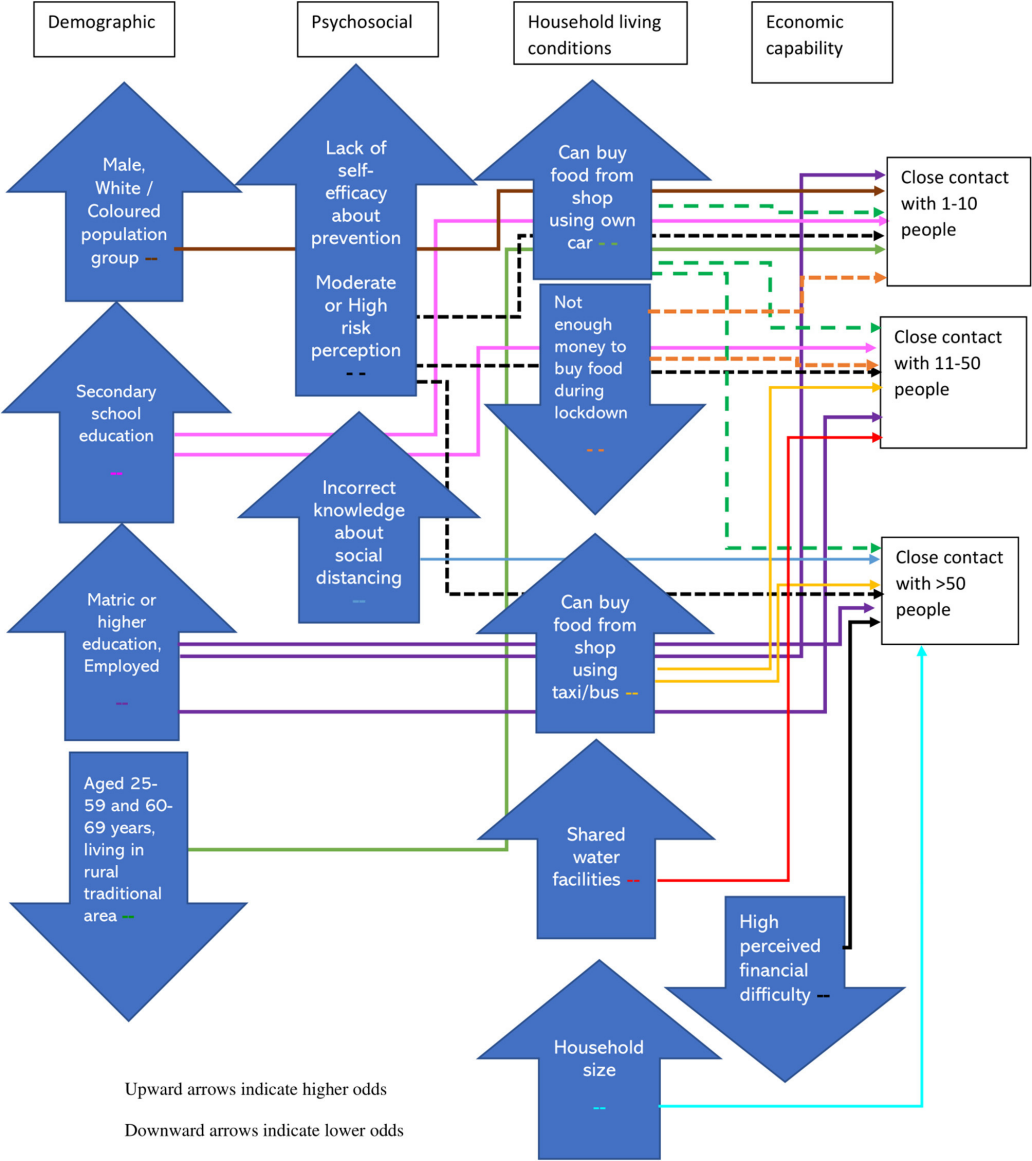
Factors associated with higher and lower odds of 1–10, 11–50, and >50 close contacts during the lockdown in South Africa.

In Figure 1 we summarise the key findings presented in Table 3. The analyses highlight 8 broad indicators within demographic, psychosocial, household living conditions and economic capabilities as being associated with numbers of close contacts.

**Table 3:** Multiple multinomial regression showing factors associated with being in contact with 1–10, 11–50 and >50 people when outside the home.

	**1–10 people**		**11–50 people**		**>50 people**	
	**OR**	**95% CI(OR)**	**OR**	**95% CI(OR)**	**OR**	**95% CI(OR)**
**Did not leave home (base category)**						
**Sociodemographic variables**						
Gender						
Female	ref	–	ref	–	ref	–
Male	1.37^**^	(1.18–1.58)	1.15	(0.96–1.37)	0.99	(0.8–1.24)
Other	1.01	(0.61–1.67)	1.18	(0.66–2.09)	1.04	(0.46–2.32)
Age group (years)						
18–24	ref	–	ref	–	ref	–
25–59	0.68^**^	(0.56–0.83)	0.82	(0.65–1.03)	0.79	(0.58–1.08)
60–69	0.58^*^	(0.4–0.84)	0.89	(0.58–1.36)	0.77	(0.43–1.37)
≥70	0.56	(0.32–1.01)	0.79	(0.38–1.65)	0.61	(0.23–1.63)
Population group						
African	ref	–	ref	–	ref	–
White	1.86^**^	(1.41–2.47)	1.13	(0.82–1.55)	0.74	(0.5–1.09)
Coloured	1.69^**^	(1.33–2.15)	1.2	(0.91–1.59)	0.88	(0.6–1.29)
Indian/Asian	0.99	(0.72–1.36)	1.08	(0.76–1.54)	0.91	(0.59–1.42)
Community of residence						
City	ref	–	ref	–	ref	–
Suburb	1.15	(0.89–1.49)	1.33	(0.98–1.8)	0.99	(0.65–1.5)
Township	0.8	(0.62–1.04)	1.3	(0.95–1.79)	1.29	(0.85–1.95)
Informal settlement	0.87	(0.6–1.26)	1.18	(0.75–1.84)	1.54	(0.82–2.88)
Rural (Traditional tribal area)	0.57^**^	(0.42–0.76)	1.06	(0.74–1.53)	1.22	(0.78–1.91)
Farm	0.99	(0.59–1.67)	1.25	(0.71–2.2)	0.85	(0.31–2.35)
Highest educational level						
Less than secondary	ref	–	ref	–	ref	–
Secondary	1.51^*^	(1.03–2.21)	1.63^*^	(1.04–2.56)	1.56	(0.86–2.82)
Matric or higher	2.35^**^	(1.64–3.37)	2.37^**^	(1.52–3.7)	1.68^*^	(1–2.81)
Employment						
Unemployed	ref	–	ref	–	ref	–
Employed full time	1.53^**^	(1.27–1.84)	1.84^**^	(1.47–2.29)	2.59^**^	(1.94–3.47)
Employed informal/part time	1.44^*^	(1.14–1.82)	1.54^*^	(1.18–2.02)	1.83^*^	(1.28–2.61)
Student	0.89	(0.69–1.15)	1.03	(0.76–1.39)	1.49	(0.98–2.28)
Self employed	1.32^*^	(1.01–1.71)	1.48^*^	(1.08–2.04)	2.14^*^	(1.38–3.32)
**Psychosocial factors**						
Incorrect knowledge that staying away from people who are infected can prevent COVID-19 infection	1.15	(0.72–1.84)	1.31	(0.76–2.27)	2.11^*^	(1.12–3.97)
Incorrect knowledge that staying 2 metres away from another person can prevent COVID-19 infection	1.35	(0.89–2.04)	1.4	(0.92–2.14)	1.91^*^	(1.13–3.25)
Thought that the lockdown was unnecessary and were angered by it	0.85	(0.69–1.05)	0.78	(0.61–1.01)	1.23	(0.91–1.65)
Self-efficacy in preventing one's self from COVID-19 infection						
Agree	ref	–	ref	–	ref	–
Unsure	1.78^**^	(1.39–2.28)	2.5^**^	(1.92–3.26)	2.40^**^	(1.74–3.31)
Disagree	1.18	(0.78–1.79)	1.83^*^	(1.17–2.85)	2.39^**^	(1.47–3.9)
Risk perception						
Low	ref	–	ref	–	ref	–
Moderate	1.55^**^	(1.28–1.88)	2.02^**^	(1.63–2.52)	1.87^**^	(1.41–2.48)
High	1.21^*^	(1.01–1.45)	1.58^**^	(1.28–1.94)	2.72^**^	(2.1–3.52)
**Household living conditions**						
Ability to easily get food to one's household during the lockdown						
Can buy from a shop within walking distance from my house	ref	–	ref	–	ref	–
Can buy from a shop, which I reach using a taxi/bus (public transport)	1.08	(0.86–1.36)	1.33^*^	(1.03–1.72)	1.83^**^	(1.33–2.53)
Can buy from a shop, which I reach using my car	1.27^*^	(1.04–1.54)	1.51^**^	(1.2–1.9)	1.42^*^	(1.04–1.95)
Do not have enough money to buy food during the lockdown	0.78^*^	(0.64–0.95)	0.73^*^	(0.58–0.92)	0.91	(0.66–1.25)
Share water facilities with other households	1.12	(0.95–1.32)	1.22^*^	(1–1.47)	1.11	(0.87–1.42)
Household size	0.99	(0.96–1.02)	1.02	(0.99–1.05)	1.04^*^	(1–1.08)
**Economic capability**						
Perceived COVID-19 related financial difficulty						
Low	ref	–	ref	–	ref	–
Moderate	1.1	(0.89–1.37)	0.82	(0.64–1.05)	0.78	(0.58–1.05)
High	1.08	(0.86–1.38)	0.79	(0.61–1.03)	0.71^*^	(0.51–0.98)

* *p < 0.05*,

** *p < 0.001*.

#### Characteristics of Those Who Came Into Close Proximity With 1–10 People

The odds of coming into close proximity with 1–10 people outside the home compared to having not come into contact with anyone were significantly higher among males than females (AOR = 1.37, 95% CI: 1.18–1.58); White [AOR = 1.86 (1.41–2.47)] and Coloured [AOR = 1.69 (1.33–2.15)] than African population groups; participants with secondary school education and who completed secondary school [AOR = 1.51 (1.03–2.21) and AOR = 2.35 (1.64–3.37)] than those with less than secondary school education; full-time employees, self-employed participants and those employed informally or part-time than unemployed participants [AOR = 1.53 (1.27–1.84), AOR = 1.32 (1.01–1.71) and AOR = 1.44 (1.14–1.82), respectively]; participants who were unsure about their self-efficacy in protecting themselves from infection than those with high self-efficacy [AOR = 1.78 (1.39–2.28)]; participants with moderate and high risk perception than low risk perception [AOR = 1.55 (1.28–1.88) and AOR = 1.21 (1.01–1.45)]; and participants who travelled to shops by their own car [AOR = 1.27 (1.04–1.54)] than by walking there.

The odds of coming into close proximity with 1–10 people compared to having not come into contact with anyone were significantly lower for 25–59 year olds [AOR = 0.68 (0.56–0.83)] and 60–69 year olds [AOR = 0.58 (0.40–0.84)] than younger people aged 18–24 years; residents of rural traditional areas [AOR = 0.57 (0.42–0.76)] than city dwellers; and participants who were unable to afford food during the lockdown than those who walked to shops to buy food [AOR = 0.78 (0.64–0.95)].

#### Characteristics of Those Who Came Into Close Proximity With 11–50 People

The odds of coming into close contact with 11–50 people was significantly higher for participants with secondary school education [AOR = 1.63 (1.04–2.56)] and who completed secondary school [AOR = 2.37 (1.52–3.70)] than those with less than secondary school education; full-time employees, self-employed participants and those employed informally or part-time [AOR = 1.84 (1.47–2.29), AOR = 1.48 (1.08–2.04), and AOR = 1.54 (1.18–2.02), respectively]; participants who were unsure or disagreed about having self-efficacy to protect themselves from infection than those with high self-efficacy [AOR = 2.50 (1.92–3.26) and AOR = 1.83 (1.17–2.85)]; participants with moderate and high risk perception than low risk perception [AOR = 2.02 (1.63–2.52) and AOR = 1.58 (1.28–1.94), respectively]; participants who travelled to shops by public transport [AOR = 1.33 (1.03–1.72)] or by their own car (AOR = 1.51 (1.20–1.90)] than those who travelled to shops by walking; and participants who lived in households that share water facilities with other households [AOR = 1.22 (1.00–1.47)]. The odds of coming into close contact with 11–50 people was significantly lower for participants who were unable to afford food during the lockdown [AOR = 0.73 (0.58–0.92)] than those who travelled to shops by walking.

#### Characteristics of Those Who Came Into Close Proximity With >50 People

The odds of coming into close contact with >50 people compared with having not come into contact with anyone was significantly higher for participants who completed secondary school than those with less than secondary school education [AOR = 1.68 (1.00–2.81)]; full-time employees, self-employed participants and those employed informally or part-time than unemployed participants [AOR = 2.59 (1.94–3.47), AOR = 2.14 (1.38–3.32), and AOR = 1.83 (1.28–2.61), respectively]; those with incorrect knowledge that staying away from infected people was a preventive measure [AOR = 2.11 (1.12–3.97)]; those with incorrect knowledge that staying 2 metres away from other people was a preventive measure [AOR = 1.91 (1.13–3.25)]; participants who were unsure or disagreed about having self-efficacy to protect themselves from becoming infected than those with high self-efficacy [AOR = 2.4 (1.74–3.31) and AOR = 2.39 (1.47–3.9)]; participants with moderate and high risk perception than low risk perception [AOR = 1.87 (1.41–2.48) and AOR = 2.72 (2.10–3.52)]; participants who travelled to shops by public transport [AOR = 1.83 (1.33–2.53)] or by their own car [AOR = 1.42 (1.04–1.95)] instead of walking; and those with higher household sizes [AOR = 1.04 (1–1.08)]. The odds of coming into close contact with >50 people were significantly lower for participants with high perceived financial difficulty as a result of the lockdown than those with low financial difficulty [AOR = 0.71 (0.51–0.98)].

## Discussion

The study provides evidence on the extent of social distancing, assessed by the number of people that participants came within close proximity to, on the last occasion that they were outside their homes, during the early days of the COVID-19 lockdown in South Africa. It provides the first evidence of psychosocial and environmental determinants associated with social distancing in the country. A fifth of South Africans reported that they were not in close proximity (within 2-metres) to others outside their homes because they had not left home. More than half were in close proximity to up to 10 people and 29% said they were in close proximity to more than 10 people. The findings provide perspective on the effectiveness of state implemented lockdown orders, which have thus far been dominated by studies in high income countries (9, 37). Abrupt social distancing regulations caught many people off guard, as there were just under 5,000 cases reported between 4th March and 27th April 2020, at the time of the lockdown enforcement (38).

The findings must be interpreted within the context of the South African lockdown where all citizens were required to stay at home, unless they needed to leave home to access essential services (39). Essential service providers and public transport were mandated to implement protocols that ensured that their patrons kept at least one square metre apart and reduce 50% of their capacity, respectively. The survey did not ask about the types of activities the participants were engaged in or request the reasons for coming into close contact with people outside their homes.

However, a review of Google's COVID-19 Community Mobility Reports, which tracked how visits and duration of time spent at various locations changed over time among users of Google Maps, provides some insight. These reports indicated that during the lockdown there was over 70% reduction in mobility in retail and recreation locations and in transit stations, over 60% reduction in workplace mobility but only a reduction of 40% in grocery and pharmacy locations (40). Larger reductions in mobility were observed in the Western Cape, Gauteng and Kwa-Zulu Natal provinces, which have large industrial urban areas, larger economies and had greater numbers of cases (41). Similarly, another national study in July 2020 found that while many South Africans reported avoiding large groups, only 25% practised physical distancing (42).

At the time of the survey, face masks were newly introduced and only became compulsory to be worn in public on 29 April 2020 (43). Our study did not ask participants if they had worn a mask when outside their homes. Visits to essential service providers such as grocery stores and pharmacies increase the chances of close proximity to people, depending on the environments in which these activities occur. For example, it is likely that travelling to, queuing outside and shopping in a crowded township mall would result in contact with more people than at a quieter suburban shopping centre. However, participants who were in close proximity of over 50 people can be seen as having not avoided, or been able to avoid, crowds. These individuals were least compliant with social distancing regulations, and could have contributed to the rapid community transmission rates.

It is, therefore, within this context, that differing patterns and strengths of association with the determinant variables were observed for those who came into contact with smaller numbers of people vs. those who were in contact were in contact with large numbers of people. Murphy et al. (44) show that various factors are linked to compliance with laws affecting freedom of movement. However, Bish and Michie (45) found few studies aimed at understanding avoidant behaviours, in their review of demographic and attitudinal determinants of behaviours during a pandemic.

Male gender, younger age and being in the White and Coloured population groups were associated with being in contact with 1–10 people and not with larger numbers of contacts. Notably, larger household size and a lack of knowledge about the importance of social distancing were associated with being in contact with more than 50 people. Employment, having at least secondary school education, lack of self-efficacy in being able to protect oneself from infection, and moderate or high risk perception of becoming infected, were all linked to increased odds of close contact with other people, where there were signs of a dose response relationship, in that the strengths of associations were higher for having >50 and 11–50 close contacts than with having 1–10 contacts.

Additionally, people who could travel to shops using their own vehicles were more likely to be in contact with others relative to remaining at home, and those who travelled by minibus taxis or other public transport were more likely to be in contact with over 10 people. Individuals who shared water facilities with other households, such as communal taps and water tanks, came into contact with 11–50 people more often than those with their own water facilities.

In agreement with the finding that males came into contact with 1–10 people more often than females, other studies have shown that men were less likely to comply with public health precautions, including hand washing and social distancing (46–48). The lower adherence to preventive measures among men may be explained by socially constructed behaviours relating to masculinity, such as masking of fear and the tendency to downplay risk (45, 49). Gender differences in labour market participation, work arrangements and household roles also determine the extent of being able to stay at home during lockdowns (50). In congruence with current findings on age effects, studies in the United States and Germany also reported less social distancing among young people (27, 51). Youth tend to have more social contacts than older people (52, 53) and in sub-Saharan African countries large multigenerational households can increase risk transmission between young and old (29). Understanding young people's motivating factors for engaging in social distancing, such as increased social responsibility (54), will inform strategies to increase social distancing among youth.

Other studies also found lower engagement in COVID-19 preventive behaviours, including social distancing, among individuals with low self-efficacy and low knowledge about preventive behaviours (26, 55, 56). Individuals with poor knowledge about the role of distancing in preventing infection were twice as likely to have over 50 close contacts, which suggests the need for public health communication to explain the mechanisms of viral transmission and thereby provide a clear rationale for distancing behaviours. Public health communication should enhance self-efficacy by providing practical solutions to perceived barriers of distancing behaviour.

While increased risk perception generally increases protective behaviours (57), in this study, individuals with high risk perceptions had more close contacts. Further research is required to understand this association. Perceived fatalism of COVID-19 has been shown to be associated with lower intention to practise protective behaviours such as social distancing (58). Additionally, coming into close contact with others is not always autonomous but could be dependent on the circumstances that allow for social distancing. Other studies argue that the perceived risk and behaviour relationship cannot be fully examined in cross sectional studies because one's current risk perception can be reflective of their risk behaviours over time (59).

Individuals with secondary or higher levels of education and those who were employed either full time, informally, part-time or self-employed were more likely to have close contact with others, because those with less than secondary school education and the unemployed reported higher rates of staying at home. Individuals with low education have previously been shown to be more compliant with preventive measures during disease outbreaks (60). Employed individuals are more often the breadwinners of the family and would have been expected to go out during lockdown for activities such as grocery shopping or to work in essential services. Self-employed and informal sector employees such as market sellers or maintenance services may have been informally seeking work during lockdown, which increased their likelihood of contact with large numbers of people. Bish and Michie (45) found the relationship of educational level and avoidant behaviour, such as avoiding large crowds, to be unclear but there was evidence of more educated people complying with avoidant behaviour.

Factors indicative of lower socio-economic living conditions, that is communal water sources, large households, and using public transport to shops were all associated with high numbers of close contacts. Residents of densely populated neighbourhoods and those who use minibus taxis or buses have a higher frequency of close contacts (52) because social distance is constrained in these environments (61–63). It is critical that preventive measures for public transport are adhered to, including disinfecting surfaces in public transport and maintaining safe distances between commuters in minibus taxi rank queues. Contrastingly, individuals who travelled to shops with their own vehicle, which is usually indicative of affluence, were also more likely to have close contacts. Having a private vehicle provides the opportunity for increased mobility, which is in turn leads to increased probability of contacts.

The determinants associated with non-compliance with social distancing can inform the development of tailored health promotion and communication strategies. As differential risks of exposure are considered for preferentially vaccinating individuals, so too should heterogeneity of group risks be considered when designing interventions. Using an intervention mapping approach, health educators can tailor and target health education messages for subgroups of individuals that were less likely to comply with social distancing regulations, such as males, young people, individuals in densely populated areas with shared water sources, the employed, and taxi commuters. Information campaigns need to improve individuals' knowledge of social distancing as a preventive measure, thereby enhancing cooperation to comply with public health advice. Campaigns should reiterate the combined effect of mask wearing, reducing gatherings, distancing and hand hygiene, particularly as fatigue in practising these behaviours sets in. Intervention development efforts need to recognise that distancing behaviours are due to willingness and perceived control but are also dependent on circumstantial feasibility of distancing. From a policy perspective, enabling environments therefore need to be created to enhance individuals' self-efficacy to protect themselves from infection and promote social distancing. Measures being implemented in several countries include home delivery of essential services, chronic medication, food parcels and social grants; temporary sites for people unable to quarantine at home (29); and enforcement of 1–2 metre distance marks in queues, shops and transit stations. This is particularly relevant because many South Africans, particularly pensioners, waited in long queues to collect social grants or food parcels during lockdown. Other policy directives include investing in better infrastructure such as sanitation, water, housing, and ventilation, as well as better infrastructure for public transport. Regulations for public transport need to be reviewed and enforced, including disinfecting surfaces and distancing protocols for commuters. Distancing and infection control protocols need to be strengthened and enforced in workplaces and areas where informal sector work is common, like streets with street vendors. Given that South Africa's second epidemic wave emerged after public events and mass gatherings during the festive season in December 2020, the regulations for gatherings needs to be reviewed and enforced.

Health communication needs to include simplified and language-appropriate targeted messages to change health behaviour and social norms, increase public accountability, and guide individuals in crowded living conditions on how to effectively social distance when outside their homes. Interventions that are community-led are more likely to increase public support for and adoption of social distancing. Notably, ways to maintain social connexion should be considered when promoting social distancing, because distancing behaviours during the pandemic have led to a decline in social connexions that are linked to poor mental health and increased desire for material wealth (64).

Finally, the unemployed, those who were unable to access or afford food and those with highest perceived financial difficulty had the highest prevalence of staying at home. Although these very poor communities adhered to lockdown regulations, they experienced severe economic impacts of the lockdown including loss of income and hunger (65). The unemployed, elderly and uneducated often rely on the economic activity of others in the household, who cannot afford to stay home and waive their means to earn an income. The South African COVID-19 lockdown is viewed as having intensified the country's pre-existing inequalities and inequities (29, 41). Lockdowns should assist those living in financial hardship in terms of service provision, economic enablement, mental health support services, infrastructure to increase living spaces, and health education so that their time spent under lockdown is more manageable.

A limitation of this study is that adherence to social distancing and other behaviours were self-reported, which is subject to recall and social desirability bias. Secondly, online surveys introduce selection bias as individuals who utilise the internet and smart phones are more likely to complete online surveys. To minimise the impact of this limitation, the online surveys were supplemented with telephonic interviews that purposely targeted individuals in poorer and high-density areas, and the data was benchmarked to the general population to increase generalisability of the findings. Thirdly, risk perception and self-efficacy were measured by single items instead of scales. Fourth, other potential mediating psychosocial variables such as social norms, perceived loss of control and perceived safety climate were not measured in this study. Strengths of the study include the rapid online survey method that provides real-time results as the COVID-19 pandemic continues. The study reports on a wide range of determinant variables from a large population-based sample. It highlights the important role of the social determinants of health in social distancing compliance in South Africa.

## Data Availability Statement

The raw data supporting the conclusions of this article can be made available from the lead author upon reasonable request.

## Ethics Statement

The studies involving human participants were reviewed and approved by the Human Sciences Research Council Research Ethics Committee. Written informed consent for participation was not required for this study in accordance with the national legislation and the institutional requirements.

## Author Contributions

RS conceptualised the paper, conducted the analysis, and led the writing. MM, IN, and TM contributed to the analysis. SPR conceived the study. ND, NV, MM, SPR, and ASD contributed to interpreting results and drafting the manuscript. All authors provided critical review of the manuscripts draughts and approved the final manuscript before submission.

## Conflict of Interest

The authors declare that the research was conducted in the absence of any commercial or financial relationships that could be construed as a potential conflict of interest.
