# Supplementary material for: Determinants of Social Distancing Among South Africans From 12 Days Into the COVID-19 Lockdown: A Cross Sectional Study
Source: Front Public Health. 2021 May 24;9:632619. doi: 10.3389/fpubh.2021.632619 (PMC8180596; doi:10.3389/fpubh.2021.632619)
Supplement: Supplementary file 1 [file Table_1.DOCX]

**Supplementary Table 1. Question items and response categories**

DEMOGRAPHIC CHARACTERISTICS

How old were you at your last birthday? (in years)

Do you subscribe to any of the following groups?

1 Female

2 Male

3 Other

To which population group do you belong?

1 Black African

2 White

3 Coloured

4 Indian/Asian

What best describes the type of community you reside in?

1 City

2 Suburb

3 Township

4 Informal settlement

5 Rural (Traditional tribal area)

6 Farm

What is the highest educational level that you have obtained?

0 None

1 Primary

2 Secondary

3 Matric

4 Tertiary

How would you describe your present employment situation?

1 Employed – full time (fixed salary per month)

2 Employed – informal sector/ part time (non-fixed salary per month)

3 Unemployed

4 Home Duties (not looking for work)

5 Full-time Student

6 Retired

7 Self Employed

KNOWLEDGE

1

I can prevent myself from becoming infected with the Coronavirus (COVID-19) by:

- Staying away from people who are infected

1 Yes

2 No

3 Don’t know

- Staying 2 meters away from another person

1 Yes

2 No

3 Don’t know

SELF-EFFICACY

To what extent do you agree or disagree with the following statement?

I am confident that I can prevent myself from getting COVID-19 virus

1 Strongly agree

2 Agree

3 Neutral

4 Disagree

5 Strongly disagree

PERCEIVED RISK

How do you rate your PERSONAL RISK of contracting the Coronavirus (COVID-19)?

1 Very high risk

2 High risk

3 Moderate risk

4 Low risk

5 Very low risk

PHYSICAL DISTANCING

The last time you were away from home, how many people did you come into close contact with? (Within 2 metres). If you are not sure, please make your best guess.

1) 1 to 3 people

2) 4 to 10

3) 11 to 20

4) 21 to 50

5) More than 50 people

6) Have not left home

ACCESS TO FOOD AND WATER

Can you get food to your household easily during the lockdown?

1 We can buy from a shop within walking distance from my house

2 We can buy from a shop, which I reach using a taxi/bus (public transport)

3 We can buy from a shop, which I reach using my car

4 We do not have enough money to buy food during the lockdown

During the current lockdown period, what is the MAIN source of water for your household to wash your hands?

1 Piped into dwelling

2 Piped into site/yard

3 Public tap

4Water from open well

5 Water from covered well or borehole

6 Surface water spring

7 River/stream

8 Water truck carrier/tanker

9 Community Water Tanks

Do you share these water facilities with other households?

1 Yes

2 No

HOUSEHOLD SIZE

How many people live in your household? (Please indicate the number, including yourself)

PERCEIVED COVID-19 RELATED FINANCIAL DIFFICULTY

To what extent do you agree or disagree with the following statements?

- I feel that the Coronavirus lockdown is making it difficult to earn my income:

1 Strongly agree

2 Agree

3 Neutral

4 Disagree

5 Strongly disagree

- I feel that the Coronavirus lockdown is making it difficult to keep my job

1 Strongly agree

2 Agree

3 Neutral

4 Disagree

5 Strongly disagree

- I feel that the Coronavirus lockdown will make it difficult to feed my family

1 Strongly agree

2 Agree

3 Neutral

4 Disagree

5 Strongly disagree

- I feel that the Coronavirus lockdown will make it difficult to pay my bills/debts

1 Strongly agree

2 Agree

3 Neutral

4 Disagree

5 Strongly disagree
